# Single-Cell Sequencing and Its Applications in Liver Cancer

**DOI:** 10.3389/fonc.2022.857037

**Published:** 2022-04-21

**Authors:** Binle Tian, Qi Li

**Affiliations:** Department of Oncology, Shanghai General Hospital, Shanghai Jiao Tong University School of Medicine, Shanghai, China

**Keywords:** single-cell sequencing, liver cancer (LC), heterogeneity, tumor microenvironment, oncogenesis, metastasis

## Abstract

As one of the most lethal cancers, primary liver cancer (PLC) has high tumor heterogeneity, including the heterogeneity between cancer cells. Traditional methods which have been used to identify tumor heterogeneity for a long time are based on large mixed cell samples, and the research results usually show average level of the cell population, ignoring the heterogeneity between cancer cells. In recent years, single-cell sequencing has been increasingly applied to the studies of PLCs. It can detect the heterogeneity between cancer cells, distinguish each cell subgroup in the tumor microenvironment (TME), and also reveal the clonal characteristics of cancer cells, contributing to understand the evolution of tumor. Here, we introduce the process of single-cell sequencing, review the applications of single-cell sequencing in the heterogeneity of cancer cells, TMEs, oncogenesis, and metastatic mechanisms of liver cancer, and discuss some of the current challenges in the field.

**Graphical Abstract:**
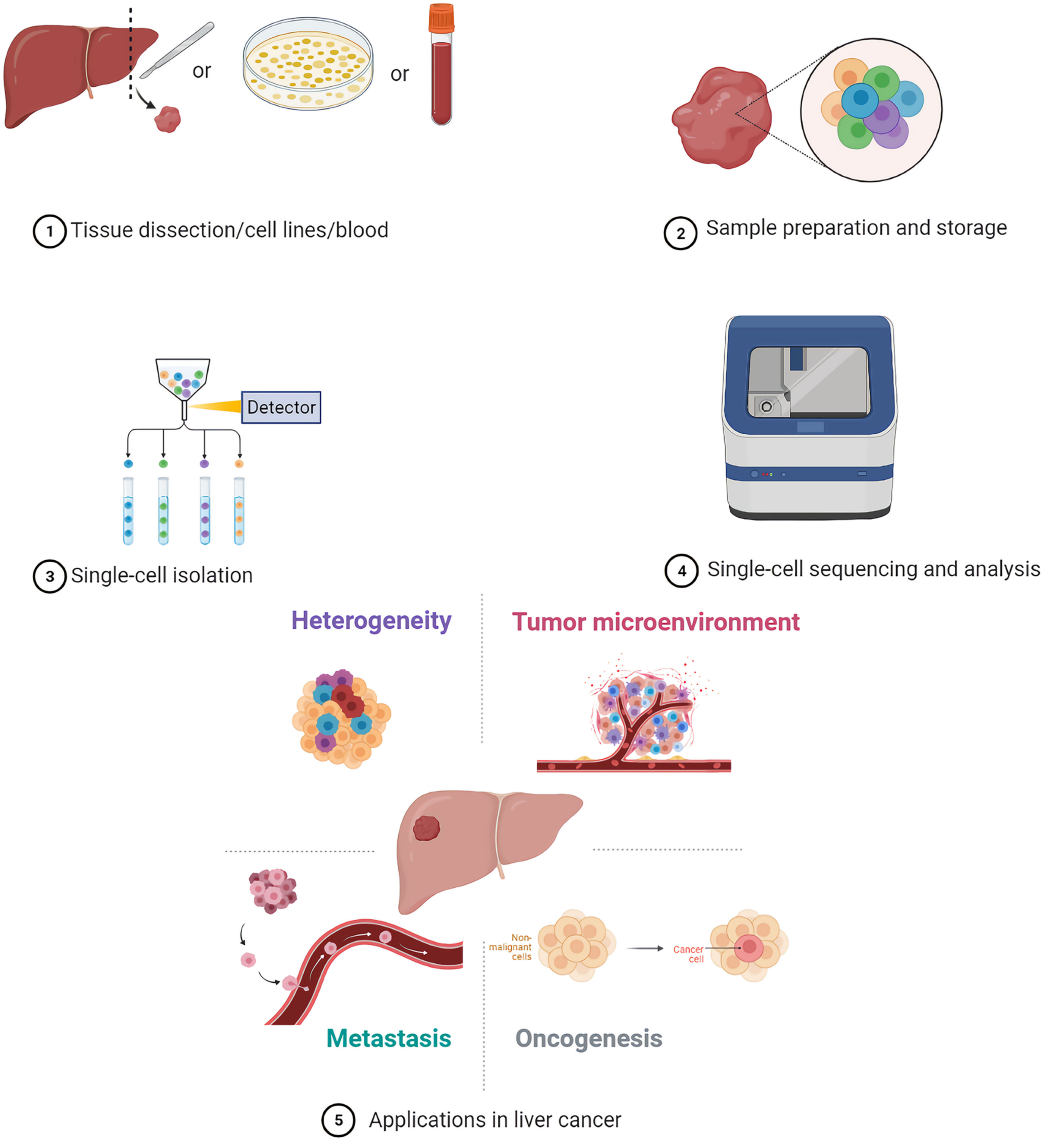


## 1 Introduction

Primary liver cancers (PLCs) are some of the most common malignancies and rank as the seventh most prevalent cancer and the third leading cause of cancer-related deaths in the world (1). PLCs comprise a heterogeneous group of malignant tumors with different histological features and an unfavorable prognosis. Hepatocellular carcinoma (HCC) and intrahepatic cholangiocarcinoma (ICC) are the most common PLCs, while the other neoplasms account for less than 1% of cases (2). HCC and ICC can develop by transformation of mature hepatocytes and cholangiocytes, respectively (2, 3). Furthermore, mature hepatocytes can also be reprogrammed into cells that closely resemble biliary epithelial cells and result in the development of ICC (4). The heterogeneity of PLCs is an important factor affecting the prognosis of patients (5). Conventional molecular profiling which is based on an average molecular phenotype from a large population of cells has been used to identify tumor heterogeneity for a long time (6). However, traditional methods such as the one mentioned above, are based on large mixed cell samples, and their results usually show the average level of cell population, whilst disregarding the heterogeneity between cells (7). In addition, it is difficult to detect rare cancer stem cells (CSCs) and circulating tumor cells (CTCs) which play important roles in tumor recurrence and metastasis using traditional sequencing methods (8, 9). In recent years, single-cell sequencing technologies have been increasingly applied in researches of liver cancer. They are used to analyze the genomics, transcriptomics, and epigenetics of tumor cells, tumor stem cells, individual cells in the tumor microenvironment (TME), and CTCs. The use of single-cell sequencing can detect heterogeneity between tumor cells and the clonal characteristics of each cell, as well as distinguish cell subgroups in the TME, thus, helping researchers understand the mechanisms of tumor evolution.

## 2 Overview of Single-Cell Sequencing Technology

Single-cell sequencing methods are mainly divided into genome, transcriptome, epigenome, and triple omics sequencing (10). The first step for single-cell sequencing is single-cell isolation, followed by multi-omics analyses (11).

### 2.1 Sample Preparation and Storage

In clinical research, it is difficult to perform single-cell sequencing of fresh samples instantly due to the long distances between clinics and laboratories in some areas. Hence, clinical samples need to be handled or stored carefully after collection to preserve their molecular integrity until molecular phenotyping is performed. Cryopreservation and methanol fixation are common methods for temporary storage of samples for single-cell sequencing applications. Warm ischemia induced by multiple stresses such as decreased oxygen supply, temperature variations and mechanical and structural stress may lead to the alteration of sample quality. Timely cryopreservation of samples after resection contributes to avoiding warm ischemia and protecting RNA quality and integrity (12). Cryopreservation may result in crystallization and disruption of cellular membranes; therefore, cryoprotectants such as dimethyl-sulfoxide are usually added to ensure the integrity and vitality of cells (13). Unlike aldehydes, methanol is a coagulating fixative that does not chemically modify nucleic acids. Alcohols can act as dehydrating agents; in the presence of salts and alcohol content that is higher than 65%, nucleic acids occur in a collapsed state but can be reverted to their original form through rehydration (14, 15). Notably, methanol fixation is challenging in tissues with a high content in proteases and RNases, such as pancreas, gall bladder, skin, lymphatic, and immune tissues (16). Interestingly, there are studies that show that methanol fixation and cryopreservation of cells and tissues would not lead to significant differences in single-cell transcriptome profiles, comparing with fresh cells (13, 16). One of the challenges to the clinical utility of CTCs is their inherent fragility, which makes these cells very unstable during transportation and storage of blood samples (17). In an evaluation of collection devices with three preservative reagents, K_3_EDTA, Cell-Free DNA BCT (BCT), and CellSave (Cellsearch), results showed that BCT and CellSave can provide the best conservation of CTCs, and BCT also provides the better conservation of RNA in comparison with K_3_EDTA (17). A few studies thus far have explicitly dealt with cell preservation protocols for single-cell sequencing (16). Further development and evaluation of cell preservation protocols are necessary to enable wider application of single-cell sequencing based on clinical samples (6).

### 2.2 Single-Cell Isolation

At present, several well-established methods can be used to isolate single cells, including serial dilution, micromanipulation, laser capture microdissection (LCM), microfluidics, fluorescence-activated cell sorting (FACS), magnetic-activated cell sorting (MACS), and the CellSearch system (18–25). FACS is one of the most common single-cell isolation methods which uses a laser beam to excite the target cells marked by a fluorescent probe, and then uses an optical detector to sort these cells according to the fluorescent signal (20). For instance, cancer cells are defined as CD31^−^/CD45^-^/7-AAD^-^ cells, and CSCs are defined as CD24^+^/CD133^+^/epithelial cell adhesion molecule (EpCAM)^+^ cells after immunofluorescence staining and sorting by flow cytometry in some single-cell analyses of liver cancer (5, 26–28). By using a cell sorting flow cytometer, this high throughput method can accurately and sensitively separate cells according to their sizes, granularities, fluorescence signals, and other features. However, the disadvantages of FACS include its complexity, over-stimulation of cells, and requirement of a large sample size and highly trained operators (19). Serial dilution is a low-cost and easy-to-operate technique which obtains single cells by continuous dilution of samples, but errors such as loss of cells during the isolation process can occur, and it is difficult to isolate specific types of cells (29). Another isolation technique, micromanipulation, is a simple and convenient method, which uses glass capillaries or patch clamps to isolate cells under the microscope. However, this method is low throughput and needs highly skilled operators, which limits its use (19). Of note, LCM can isolate target (individual) cells from mostly solid tissue samples by laser cutting and its most prominent advantage is its ability to separate cells accurately without destroying the integrity of adjacent tissues. Due to the requirement of selecting target cells, one disadvantage of LCM is the need of experienced operators (30). Microfluidic technologies can also isolate single cells based on three operating principles: microstructures, hydrodynamic effect-based methods, and droplet-based assays. This method has become increasingly popular for single-cell studies because it can provide precise fluid control and low sample consumption (19, 29). In contrast, MACS uses antibodies, enzymes, lectins, or streptavidins combined with magnetic beads to bind specific proteins on the surface of target cells. When the cell mixture is placed in an external magnetic field, the magnetic beads are activated, and the labeled cells will polarize while other cells are washed out. The target cells can then be collected after the magnetic field is removed (19). MACS technology has high specificity and causes little damage to cells, but it can only utilize cell surface molecules as markers for isolation of live cells (19). The low frequency and heterogeneity of CTCs makes their detection extremely difficult (31). The CellSearch system is the first and only Food and Drug Administration (FDA)-cleared method designed for the enumeration of CTCs from peripheral blood. This automated system utilizes immunomagnetic capturing of cells that express EpCAM on the cell surface (32). Therefore, one of the disadvantages of the CellSearch system is that this method is limited by its reliance on the enrichment of EpCAM on the cells surface and thus, only the EpCAM-positive subpopulation of CTCs are detected while CTCs lacking EpCAM expression would be invisible in the CellSearch system (33). The advantages and limitations of the above-mentioned methods are briefly summarized in **Table 1**.

**Table 1:** Single-cell isolation methods.

Methods	Advantages	Disadvantages	References
Serial dilution	Simple process; low cost	Isolation errors; loss of cells	(29, 34, 35)
Micromanipulation	Visible operation; simple process; low cost	Skilled personnel needed; low throughput; susceptible to errors	(36–39)
Laser capture microdissection	Intact fixed and live tissue	Skilled personnel needed; low throughput; biased selection	(30, 40–42)
Microfluidic	High accuracy; low sample consumption	Often restricted to one single application; high cost	(43–47)
Fluorescence-activated cell sorting	High specificity; multiple parameters	Large amount of material needed; severe cell damage	(20, 48, 49)
Magnetic-activated cell sorting	High specificity; little cell damage	Only cell surface molecules can be used as makers	(50–53)
CellSearch system	Enumeration and capture of CTCs; high throughput	Biased toward markers used for isolation; high cost	(31–33, 54)

### 2.3 Sequencing Techniques

A single cell contains only 6 to 12 pg of DNA and 10 to 60 pg of RNA; therefore, multiple rounds of amplification are required to increase the amount of extracted nucleic acids (DNA and RNA) and reach the detection limit (19, 55–57). We briefly summarize the methods for single-cell analysis and their advantages and disadvantages in **Table 2**.

**Table 2:** Summary of single-cell analysis methods.

	Methods	Advantages	Disadvantages	References
Genome	DOP-PCR	High throughput	Uneven amplification, low coverage, amplification errors, allele dropout	(58, 59)
	MDA	Simplicity, high fidelity, low false positive rate	Amplification bias, allele dropout	(60)
	MALBAC	High uniformity, low amplification bias	Allele dropout	(61)
Transcriptome	STRT-seq	Highly multiplexed method, pinpoint the exact location of the 5’ end of transcripts	Technical variation, cannot span the entire transcript length	(62)
	Smart-seq	Full-length coverage across transcripts	Distort the difference	(63)
	CEL-seq	High specificity, ratio fidelity	Low efficiency, reduced sensitivity for low-expression transcripts	(64)
	InDrop	High throughput, low cost	Low mRNA capture efficiency, high error rate	(65)
	Drop-seq	High throughput, low cost	Relatively low sensitivity	(66)
	10x Chromium Genomics	High throughput, high molecular sensitivity and precision, low technical noise, time saving	High cost	(67)
Epigenome	RRBS	Relatively low cost	Low throughput, low coverage	(68)
	WGBS	Low amplification bias, correct assignment of paired-end fragments	Low library complexity	(69)
	CGI-seq	High efficiency, simplified procedure	Inconsistent and/or low coverage	(70)
	ATAC-seq	High coverage, high sensitivity	Low recovery of DNA fragments	(71, 72)
	DNase-seq	Simplicity	Large amount of material needed, high error rate	(73)
	ChIP-seq	High resolution, high coverage	Highly dependent on the quality of antibody	(74)
	Drop-ChIP	High throughput, high specificity	Low coverage	(75)
Multi-omics	Trio-seq	Simultaneous analyses of genome, epigenome, and transcriptome in the same single cell	Low throughput	(76)
	CITE-seq	Providing additional phenotypic information, high compatibility	Only cell surface protein can be characterized	(77)
	10x multiome ATAC+RNA	Powerful capability to characterize cellular diversity, high accuracy	Low compatibility	(78)

DOP-PCR, degenerate oligonucleotide-primed polymerase chain reaction; MDA, multiple displacement amplification; MALBAC, Multiple annealing and looping-based amplification cycles; STRT-seq, single-cell tagged reverse transcription sequencing; Smart-seq, switching mechanism at 5’ end of the RNA transcript sequencing; CEL-seq, cell expression by linear amplification and sequencing; InDrop, indexing droplets; RRBS, reduced representation bisulfite sequencing; WGBS, whole genome bisulfite sequencing; CGI-seq, genome-wide CpG island methylation sequencing; ATAC-seq, assay for transposase accessible chromatin sequencing; ChIP-seq, chromatin immunoprecipitation sequencing; Trio-seq, triple omics sequencing; CITE-seq, Cellular Indexing of Transcriptomes and Epitopes by sequencing.

#### 2.3.1 Single-Cell Genome Sequencing

After a target cell is isolated and the DNA is extracted, whole genome amplification (WGA) is performed. In the past, WGA was mainly achieved by polymerase chain reaction (PCR), including linker-adapter PCR (LA-PCR), interspersed repetitive sequence PCR (IRS-PCR), primer extension pre-amplification PCR (PEP-PCR), and degenerate oligonucleotide-primed PCR (DOP-PCR) (19).

In 2011, Navin et al. published the first study based on the use of DOP-PCR to apply single-cell genome sequencing to reveal the cancer genome heterogeneity (58). DOP-PCR is an exponential amplification process, such that any bias will be exponentially amplified, leading to some limitations which include uneven amplification, low coverage, and amplification errors (59, 61).

Due to its simplicity and high fidelity, multiple displacement amplification (MDA) has become the most commonly used method for WGA today. MDA is exponential like PCR, but has a higher genome coverage and lower error rate in comparison with PCR (61). However, in the initial stage of MDA, there are differences in the binding ability of different regions, which may result in over-amplification or under-amplification of different genomic regions, causing amplification bias (60).

Another new method is multiple annealing and looping-based amplification cycles (MALBAC), which is a combination of DOP-PCR and MDA, and also has the unique properties of quasi-linear amplification (61). The key point of MALBAC is that the 3′ end of the amplified product is designed with a specific primer with a complementary 5′ end. After a round of amplification, the complementary sequence tags at both ends bind to form a self-enclosed “hairpin” structure to prevent the amplified product from being copied and allow the original genomic DNA to be copied. Therefore, MALBAC is advantageous because it reduces amplification bias and increases genome coverage. The amplified genome is then sequenced and analyzed by DNA next-generation sequencing technology, and different types of genetic changes can be accurately detected.

#### 2.3.2 Single-Cell Transcriptome Sequencing

Although mRNA is not as scarce as DNA in single cells, it still needs to be amplified. The first step in whole transcriptome amplification is to reverse-transcribe the mRNA into complementary DNA, followed by PCR (79). The research methodology of mRNA transcriptomics in a single cell based on high-throughput sequencing was first reported in 2009 (80). In 2011, Islam et al. invented single-cell tagged reverse transcription sequencing which can detect various mixed cell samples such as highly heterogeneous tumor cell samples on a large scale (62).

Subsequently, Ramsköld et al. invented switching mechanism at the 5′ end of the RNA transcript sequencing (Smart-seq) that can detect the full length of mRNA in 2012 (63). This technology can significantly improve the reading coverage of the transcriptome, enabling detailed analyses of alternative splicing and identification of single nucleotide polymorphisms and other mutations. In 2014, Picelli et al. created Smart-Seq2, modifying the primers in Smart-Seq into locked nucleic acids to improve sensitivity, accuracy, and full-length coverage (81). In addition to the above PCR-based amplification methods, *in vitro* transcription methods such as the cell expression by linear amplification and sequencing technique which uses linear amplification to reduce the error rate to make sequencing more accurate can also be used (64).

In these years, many droplet-based systems for high-throughput single-cell RNA-seq have attracted attention, such as inDrop, Drop-seq, and 10x Chromium Genomics (65–67). These technologies use similar designs to generate droplets, differentiate single cells by on-bead primers with barcodes, and apply unique molecular identifier (UMI) for bias correction (82). But different methods of bead manufacturing, barcode design, and cDNA amplification lead to the difference in cost, time, cell capture efficiency, and detection sensitivity of these technologies (82, 83). The inDrop platform encapsulates single cells into droplets with lysis buffer, reverse transcription (RT) reagents, and barcoded hydrogel microspheres (84). Then, a library of barcoded hydrogel microspheres (BHMs) co-encapsulated with cells can be synthesized. After encapsulation primers are released, cDNA in each droplet is tagged with a barcode during reverse transcription. Droplets are then broken and material from all cells is linearly amplified before sequencing (65). Like inDrop, Drop-seq also requires single cell suspension and co-encapsulates each single cell with a barcoded bead in nanoliter-scale droplets (84). However, photoactivation of the oligonucleotides is unneeded in Drop-Seq. Cells are lysed after they have been isolated in droplets, and then mRNAs are capture on its companion microparticle, forming Single-cell Transcriptomes Attached to Microparticles (STAMPs). Subsequently, the STAMP barcodes are applied to infer each transcript’s cell of origin (66). The core of 10x Chromium Genomics is a Gel bead in EMulsion (GEM). RT takes place inside each GEM, after which cDNAs are pooled for amplification and library construction in bulk (67). Numerous systematic comparisons of droplet-based high-throughput single-cell RNA sequencing methods have been conducted to reveal the distinguishing features and suitable applications (82, 83, 85). In summary, 10x Chromium Genomics is reported to have the strongest consistent performance (82, 83). As a more maturely commercialized system, 10x Chromium Genomics generally requires less time but has higher molecular sensitivity and precision and less technical noise (82). One of its few disadvantages is the high price tag. By contrast, Drop-seq exhibits a significant advantage in experimental cost. Therefore, Drop-seq is a popular option for individual labs because of its balanced performance and lower cost (82).

#### 2.3.3 Single-Cell Epigenome Sequencing

In addition to the genome, the epigenome, especially DNA methylation, also plays a significant role in regulating gene expression. One method of single-cell DNA methylome sequencing is reduced representation bisulfite sequencing (RRBS) which can analyze the DNA methylation level of the whole cell (68). Furthermore, in 2015, Farlik et al. invented whole genome bisulfite sequencing which can detect DNA methylation in a small number of cell populations and single cells (69). Another single-cell DNA methylome sequencing method is genome-wide CpG island (CGI) methylation sequencing for single cells that sequences genomic regions with high CpG content, providing 72.7% CGI coverage in each cell (70).

Regions of open chromatin possibly have some gene regulatory functions. Methods of profiling open chromatin are usually based on the conception that DNA regions in the open chromatin conformation are more accessible to enzymes *in vitro*, such as Tn5 transposase in the assay for transposase accessible chromatin sequencing (ATAC-seq) and DNase I in the DNase-seq (73, 86). Single-cell assay for transposase accessible chromatin sequencing, which is a type of single-cell chromatin structure sequencing, is applied to identify functionally relevant chromatin markers among specific subpopulations of cancer cells (72).

Histone modifications contain many chemical modifications on the varied histone sites, like methylation, phosphorylation, and acetylation. Genome-wide histone modifications are commonly profiled by chromatin immunoprecipitation (ChIP) followed by the sequencing (ChIP-seq), which uses an antibody to enrich the target chromatin carrying a specific histone modification (74). Recently, Drop-ChIP has been developed based on the strategy that combines drop-based microfluidics and DNA barcoding for pooling thousands of individual cells before antibody immunoprecipitation (75).

#### 2.3.4 Single-Cell Multi-Omics Sequencing

Sequencing methods for the single-cell genome, transcriptome, and DNA methylome have been developed successively. In order to accurately analyze the mutual regulation mechanisms among the genome, transcriptome, and DNA methylome, these omics methods need to be performed in the same single cell. Therefore, Hou et al. invented single-cell triple omics sequencing (Trio-seq) in 2016, which can be used to analyze the genomic copy-number variations (CNVs), transcriptome, and DNA methylome of a single mammalian cell simultaneously (76). Cellular Indexing of Transcriptomes and Epitopes by sequencing (CITE-seq) combines highly multiplexed antibody-based detection of protein markers together with unbiased transcriptome profiling for thousands of single cells in parallel (77). Compared with single-cell RNA-seq, CITE-seq provides more detailed characterization of cellular phenotypes, in which oligonucleotide-labeled antibodies are applied to integrate cellular protein and transcriptome measurements into an efficient, sequencing-based readout of single cells. Additionally, other multi-omics sequencing methods have been developed. For instance, weighted-nearest neighbor (WNN) analysis of 10x multiome ATAC+RNA has been used to integrate multiple data types measured within a cell and to obtain a joint definition of cellular state (78). Recently, 15 gene signatures associated with the survival prognosis of HCC patients have been identified by analyzing and integrating ATAC-seq and RNA-seq (87).

### 2.4 Experimental Design for Single-Cell Sequencing

Most single-cell sequencing protocols begin by isolating single cells from cell suspensions. Preparation of high-quality single-cell suspensions is key to successful single-cell studies. Initially, tissues are disaggregated by mechanical dissociation, and then enzymatic digestion is used to separate cells. Finally, suspensions are cleaned by filtering through a mesh or strainer before capture of single cells. Although most studies use fresh samples, alternatives include cryopreservation and methanol fixation for temporary storage.

To perform any kind of single-cell sequencing assay, single cells have to be isolated from the system of interest. The method of choice to purify thousands of single cells is FACS (88). By using a cell sorting flow cytometer, this high throughput method can accurately and sensitively separate cells according to their sizes, granularities, fluorescence signals, and other features. However, FACS requires very large numbers of cells (typically tens of thousands) as starting material. Thus, micromanipulation provides an alternative approach when only a few cells are available.

A single cell contains only 6 to 12 pg of DNA and 10 to 60 pg of RNA, therefore, multiple rounds of amplification are required to increase the amount of extracted nucleic acids (DNA and RNA) and reach the detection limit. In addition, as current high-throughput sequencing platforms are only able to sequence DNA molecules, reverse transcription (from mRNA to cDNA) followed by cDNA amplification is necessary before sequencing can be performed (89). Methods for amplification, analysis, and their advantages as well as disadvantages are introduced above. Choosing appropriate strategies based on the actual situation, such as sample quality, equipment, costs and targets.

Finally, in order to interpret data of single-cell sequencing in a biological context and to assess the relevance of these data, functional validation is an essential step (89). For example, if analysis results in the identification of certain cell types characterized by certain marker genes, immunohistochemistry gene-specific fluorescence *in situ* hybridization (FISH) probes and immunohistochemical antibody staining of sample sections can be used to validate that the functional protein is present.

## 3 Applications of Single-Cell Sequencing in Liver Cancer

As a rapidly developing technology, single-cell sequencing has already been used in the research of diversified cancers, such as melanomas (90–92), colorectal carcinoma (93–96), lung (97–100), breast (101–108), and prostate cancer (109, 110). Like the above-mentioned cancers, single-cell sequencing has also been widely used for liver cancer research. Therefore, we summarize the studies that used single-cell sequencing to characterize liver cancers in **Table 3**.

**Table 3:** Single-cell sequencing in characterization of liver cancers.

Author	Tumor type	Analyses	Single-cell technology	Patients	Cells	Year	Reference
Hou et al.	HCC	Intratumor heterogeneity, subtyping, mutation profiling	Trio-seq	1	25	2016	(76)
Zheng et al.	HCC	Heterogeneity of CSCs, CSC subpopulations	10x Chromium Genomics	1	3,847	2018	(5)
Ho et al.	HCC	Intratumor heterogeneity, subtyping	RNA-seq	1	139	2019	(111)
Zhang et al.	HCC	Intertumor and intratumor heterogeneity, subtyping	RNA-seq	8	NA	2019	(112)
Ma et al.	HCC, ICC	Intertumor and intratumor heterogeneity, TME	10x Chromium Genomics	19	5,115	2019	(113)
Losic et al.	HCC	Intratumor heterogeneity	10x Chromium Genomics	2	38,553	2020	(114)
Zhang et al.	ICC	Intertumor heterogeneity, TME	10x Chromium Genomics	8	56,871	2020	(26)
Wu et al.	HCC, ICC, cHCC-ICC	Spatial heterogeneity, TME, CSC subpopulations	10x Chromium Genomics	7	NA	2021	(115)
Zheng et al.	HCC	TME, subtyping of T cells	RNA-seq	6	5,063	2017	(116)
Zhang et al.	HCC	TME, immune profiles, macrophage subsets	10x Chromium Genomics	16	77,321	2019	(117)
Liu et al.	HCC	TME, molecular profiles of T cells	10x Chromium Genomics	13	8,047	2020	(118)
Zheng et al.	HCC	Immune heterogeneity, TME, molecular profiles and distribution of DPT cells	10x Chromium Genomics, TCR-seq	13	17,432,600	2020	(119)
Li et al.	HCC	TME, subtyping of T cells	10x Chromium Genomics	15	150,000	2020	(120)
Sun et al.	HCC	TME, tumor recurrence	10x Chromium Genomics	18	16,498	2021	(121)
Ho et al.	HCC	TME, Intertumor and intratumor heterogeneity, immune heterogeneity	10x Chromium Genomics	8	43,645	2021	(122)
Duan et al.	HCC	Clonal origins, evolutionary mechanisms	WGS	3	111	2018	(27)
Xue et al.	cHCC-ICC	Clonal origins, genomic profiles	WGS	133	NA	2019	(123)
Chen et al.	HCC	Clonal origins	VCS, WGS	1	264	2019	(124)
Guo et al.	HCC	Clonal evolutions	DNA-seq, RNA-seq	14	28,975	2021	(125)
D’Avola et al.	HCC	Heterogeneity of CTCs	10x Chromium Genomics, WGS	2	10,234	2018	(126)
Sun et al.	HCC	Heterogeneity of CTCs, mechanisms of metastasis	RNA-seq	73	NA	2018	(127)
Sun et al.	HCC	Spatial heterogeneity, metastatic seeding and immune-escape mechanism of CTCs	RNA-seq	10	131	2021	(128)

HCC, hepatocellular carcinoma; Trio-seq, triple omics sequencing; CSC, cancer stem cell; RNA-seq, RNA sequencing; ICC, intrahepatic cholangiocarcinoma; TME, tumor microenvironment; DPT cells, double-positive T cells; TCR-seq, T-cell receptor sequencing; DNA-seq, DNA sequencing; WGS, whole-genome sequencing; cHCC-ICC, combined hepatocellular and intrahepatic cholangiocarcinoma; VCS: virome capture sequencing; CTC, circulating tumor cell.

### 3.1 Heterogeneity of Liver Cancer

PLCs have high heterogeneity (intertumor and intratumor heterogeneity) which is closely related to their occurrence, progression, and recurrence (129). Intertumor heterogeneity usually refers to PLCs from different patients whose variant genotypes and phenotypes are induced by diverse etiological and environmental factors. Contrastingly, intratumor heterogeneity refers to genomic and biological variations that result from the evolution of different tumor cells under the influence of various microenvironments in a tumor lesion (129, 130).

Currently, the understanding of PLC heterogeneity is mostly limited to intertumor heterogeneity, but single-cell sequencing now provides a platform for the study of intratumor heterogeneity. In 2016, Hou et al. applied single-cell Trio-seq (scTrio-seq) to 25 single cancer cells derived from an HCC tissue sample and identified two subpopulations within these cells based on the genome, transcriptome, and DNA methylome of individual cells (76). Cancer cells in the minor subpopulation had more invasive markers, were more likely to escape immune recognition, and CNV analysis indicated more copy-gain events in comparison to the cancer cells in the other subpopulation.

Zheng et al. used Smart-Seq to perform single-cell RNA sequencing (scRNA-seq) of CSCs from human liver cancer cell lines (HuH1 and HuH7) and a biopsy HCC sample, demonstrating that the CSCs of HCC exhibited biological and transcriptome heterogeneity for the first time (5). In this study, CSCs were defined as CD24^+^/CD133^+^/EpCAM^+^/CD45^-^ cells. This study showed that CSCs were phenotypically, functionally, and transcriptionally heterogeneous at the single-cell level, with distinct CSC subpopulations containing various molecular signatures. Moreover, different gene expression levels in each CSC subpopulation were independently associated with prognosis in HCC, which indicated that diverse CSC transcriptomes influenced intratumor heterogeneity and tumor progression. In another study, a rare CD24^+^/CD44^+^ cell subclone with specific oncogenic gene expression signature was identified within the EpCAM^+^ cells (111). The combination of CD24 and CD44 expression, such as CD24^+^/CD44^+^ and CD24^-^/CD44^+^, had been used to define CSCs in various other cancers, including breast, ovarian, prostate, pancreatic, colorectal, lung and renal cancer, but this had not yet been reported in HCC (131–142). These researches identified rare CSC subpopulations and explored inter-relationship between different liver CSC markers and their unique gene expression signatures by means of single-cell sequencing, providing crucial clues for the definition and annihilation of liver CSCs.

Zhang et al. performed scRNA-seq on 42 samples from eight patients with HCC and identified three different HCC subtypes based on their different immune statues, revealing the expression levels of chemokines/cytokines and the metabolic characteristics of different subtypes (112). Another study confirmed that tumors with higher transcriptomic diversity were more aggressive and were associated with worse overall survival and progression-free survival of patients (113).

In 2020, Losic et al. performed scRNA-seq using 38,553 cells from seven tumoral regions from two patients with HCC to identify tumor heterogeneity (114). HCC cells in all regions of one patient mostly belonged to one molecular class, while the HCC cells in the other patient belonged to multiple molecular classes. At the gene regulatory level, this study found profound differences in transcription factor signaling among the tested tumoral regions of the two patients, revealing significant heterogeneity in the activation status of transcription factors across distant regions within the same tumor nodule (114).

Zhang et al. performed scRNA-seq on 56,871 single cells from eight ICC tissues. This study identified various tumor, immune, and stromal cells, and divided these tumor cells into four main subclusters (0, 1, 2, and 3) based on their CNVs and DEGs; notably, a high degree of intertumor heterogeneity was observed (26). Interestingly, tumor cells in subcluster 0 were characterized by high expression levels of mesenchymal markers such as collagen type I alpha I, fibronectin, and insulin-like growth factor-binding protein 7, indicating epithelial-mesenchymal transition (EMT) characteristics. In subclusters 1 and 2, tumor cells displayed high expression levels of the malignancy-promoting factors S100P and fatty acid binding protein 5 and the immune-associated genes *CD74* and *HLA-DRA*, respectively. In subcluster 3, tumor cells from a recurrent patient displayed high expression levels of serine protease inhibitor Kazal-type 1 (SPINK1).

High-resolution spatial transcriptomes of PLCs were constructed by scRNA-seq to reveal the extensive global and local intratumor heterogeneities of tumors and TMEs (115). It was reported to be the first study to analyze the genome-wide TME characteristics from normal to leading-edge to tumor regions, identifying the vital role of complete fibrous capsule for both TME architecture and intratumor heterogeneity. Furthermore, spatial heterogeneity was proved in a very early stage of HCC (a tiny tumor nodule with a 1-cm diameter) based on the asymmetrical distribution and biological behaviors of tumor cell subpopulations.

### 3.2 TMEs of Liver Cancer

TMEs, including various types of cells and non-cellular materials, play a critical role in the occurrence, progression, and metastasis of tumors (143). There may be great distinctions between TMEs among different types of cancers and among different patients with the same type of cancer. Moreover, TMEs can constantly change as the tumor progresses. Therefore, it is impossible to accurately identify the state of a TME in theory (144). However, in some cases, the TME can be specialized to show typical traits which may affect tumor progression. Understanding the characteristics of TMEs can help to profile the crosstalk between the TMEs and cancer cells, and develop novel strategies for tumor treatment (145).

Immune cells play crucial roles in the liver cancer microenvironment and crosstalk with tumor cells. The recruitment and function of immune cells are regulated by the TMEs, and these immune cells can also affect adjacent tumor cells (144, 146). Zheng et al. performed scRNA-seq on 5,063 T cells isolated from peripheral blood, tumor samples, and adjacent normal tissues from six patients with HCC, identifying 11 T cell subsets based on their molecular and functional characteristics (116). These findings provided evidence for the differential distribution of CD8^+^ T cells as a feature in the TME of HCC. In tumor samples, the proportion of mucosal-associated invariant T cells was significantly lower than that of adjacent normal tissues. In addition, tumor-infiltrating exhausted CD8^+^ T cells and regulatory T cells (Tregs) were enriched in HCC TMEs.

In another study, the single-cell transcriptomic landscape of 5,115 single cells from 19 patients with HCC and ICC was determined by scRNA-seq (113). Subsequently, eight samples with a highly heterogeneous composition of stromal cells were selected for further analysis and divided into high-diversity and low-diversity groups based on the diversity of the tumor cells. Subsequent analysis showed that vascular endothelial growth factor (VEGF) was the most differentially expressed gene between high-diversity and low-diversity groups, especially in cancer-associated fibroblasts (CAFs), tumor-associated macrophages (TAMs), and tumor-associated endothelial cells (TECs). Furthermore, they demonstrated that VEGF took part in the regulation of hypoxia-related genes and might be a key player in the reprogramming of TMEs, providing a mechanistic rationale for the combination therapy of immune checkpoint inhibitors and anti-VEGF.

Different types of single-cell sequencing methods are powerful instruments to study the state and dynamics of different immune cells. Zhang et al. applied two different scRNA-seq technologies based on more than 75,000 CD45^+^ individual immune cells from the tumor, adjacent liver, hepatic lymph node, blood, and ascites of 16 patients with HCC to explore a sufficient resource for understanding/characterizing the immune cells in HCC (117). In this study, enrichment of two distinct macrophage states, myeloid-derived suppressor cell (MDSC)-like macrophages and tumor-associated macrophage (TAM)-like macrophages were identified in HCC tumor tissues. TAM-like macrophages in HCC were very similar to the TAMs identified in lung cancer, and highly expressed marker genes associated with poor prognosis, indicating this type of tumor-infiltrating TAMs as a possible cellular candidate for therapeutic targeting in HCC (117, 147).

Distinct fibroblast subpopulations were identified by scRNA-seq based on 2,941 high-quality fibroblasts from patients with ICC (26). Each subpopulation expressed high levels of canonical fibroblast markers and displayed distinct transcriptomic signatures. The fibroblasts in the largest subpopulation were characterized by microvasculature signature genes, such as CD146 (*MCAM*), *MYH11*, *GJA4*, and *RGS5*, and designated as vascular cancer-associated fibroblasts (vCAFs). *In vivo* and *in vitro* experiments showed that vCAFs can significantly promote the proliferation of tumor cells and tumor stem cells. These results proved novel cellular crosstalk between ICC cells and vCAFs at single-cell resolution and revealed potential therapeutic targets.

Zheng et al. described the spatial heterogeneity of the immune microenvironment in HCC (119). This study revealed that the number of CD4^+^ effector memory T cells gradually increased when moving from the nontumor region to the tumor core, while CD8^+^ effector memory T cells showed the opposite trend. Additionally, the number of Tregs decreased from the tumor core and leading-edge to the nontumor region as previously reported (148). Further analysis found that in 70% of patients with HCC, a group of double-positive T (DPT) cells with CD4 and CD8 characteristics were enriched in the leading-edge region; PD-1^+^CD45RO^+^T cells accounted for a major subpopulation of DPT cells. By applying multiplex immunofluorescence tissue staining, the enrichment of DPT cells and PD-1^+^DPT cells in the leading-edge region was confirmed once again.

Sun et al. compared the transcriptomes of 16,498 cells between primary and early-relapse HCC cases, revealing a distinct immune ecosystem in early-relapse HCC. Remarkably, they described a unique subgroup of CD8^+^T cells, with high expression of CD161, innate-like dysfunctional cytotoxicity and low clonal expansion. The enrichment of these cells was associated with a worse prognosis. Some previous studies suggested that CD161 was associated with the memory function of T cells, constituting the adaptive immune response system (149, 150). Further analysis indicated that the origin of early-relapse tumor cells was subclonal and might result in the loss of major clonal neoantigens during recurrence. Moreover, they demonstrated that CD161^+^CD8^+^T cells secreted significantly less granzyme B and showed lower clonal expansion than CD161^-^CD8^+^T cells. Therefore, early recurrence developed, regardless of the significant elevation of CD161^+^CD8^+^T cell infiltration, suggesting that these T cells are unable to prevent the intrahepatic dissemination of HCC. Interestingly, in another study, the identification and functional analysis of CD161^+^PD-1^+^CD8^+^T cells in HCC by scRNA-seq came to nearly opposite conclusions (120). This research revealed that CD8^+^PD-1^+^CD161^+^T cells was significantly decreased in tumor tissues and had stronger cytotoxicity, as well as proliferative capacity. Besides, the higher infiltration level of CD8^+^PD-1^+^CD161^+^T cells indicated better prognosis. The opposite conclusions might partially result from the difference of T cell subset with or without PD-1 positive and the distinction between primary and relapse HCC. The proportion and function of CD161^+^T cells in HCC remain controversial. Hence, further study with a larger cohort in HCC cases should be warranted. In addition, it is noteworthy that inhibitory CD161 receptor has been identified as a potential immunotherapy target in glioma-infiltrating T cells (151). The anti-tumor function of CD161 blockade in liver cancer is a subject worthy of study, and CD161 may become a promising immunotherapy target in liver cancer.

Wu et al. progressively compared the spatial TME characteristics from nontumor to leading-edge to tumor regions, demonstrating that the tumor capsule potentially affected intratumor spatial cluster continuity, transcriptome diversity, and immune cell infiltration (115). Their results revealed that the integrity of the capsule was closely related to the distribution of their surrounding stromal and immune cells but had few effects on the activities of hallmark pathways in neither normal nor tumor regions.

### 3.3 Oncogenesis of Liver Cancer

Tumorigenesis, a multi-stage complex process, is the result of diverse gene changes. Genes or cell subclusters that play crucial roles in the development of tumors can be found by single-cell sequencing, which can not only predict the potential targets for treatment, but also provide biomarkers for diagnosis and prognosis (152).

Duan et al. used single-cell whole-genome sequencing (WGS) to profile 96 tumor cells and 15 normal liver cells from three male patients with hepatitis B virus (HBV)-associated HCC and demonstrated that HCCs might be of single or multiple origins (27). A single-nodular HCC tumor with a portal vein tumor thrombus (PVTT), a multifocal HCC tumor, and a confluent multinodular HCC tumor were detected by single-cell WGS. Results showed that both single-nodular HCC with PVTT and multifocal HCC tumors were derived from a single cell that was stimulated by the HBV integration events, in view of the same HBV integration sites found in each tumor cell. On the contrary, confluent multinodular HCC tumors had multiple origins, and each tumor clone was derived from different cells harboring various clone mutation groups. In addition, they found that *ZNF717*, a potential driver gene with high mutation frequency at both single cell and population levels, acted as a tumor suppressor by regulating the interleukin 6/signal transducer and activator of transcription 3 pathway in HCC. This study reveals the multiple distinct tumor evolutionary mechanisms in HCC. These different evolution patterns lead to different HCC-specific morphologies and severely call for different treatment strategies. Accordingly, understanding cancer evolution may aid to guiding the individualized treatment in HCC.

In 2019, a large-scale integrative analysis of 133 combined hepatocellular and intrahepatic cholangiocarcinoma (cHCC-ICC) cases was performed, including single nucleus sequencing (123). In this study, nuclei were individually isolated and subjected to WGA using MALBAC, followed by sequencing and analysis. According to Allen and Lisa’s criteria, cHCC-ICC cases were divided into separate-, combined-, and mixed types (153). The results showed that separate type cHCC-ICCs had both mono- and multiclonal origins, while both combined and mixed type cHCC-ICCs exhibited monoclonal origins. Moreover, combined type cHCC-ICC showed strong ICC-like characteristics, whereas mixed type cHCC-ICC showed HCC-like characteristics. Additionally, the expression of nestin was remarkably higher in both combined and mixed type cHCC-ICCs than in separate type HCC. Although, the former two types are distinct molecular subtypes, they both showed stem-like characteristics and poor prognosis (123). Therefore, nestin was expected to replace EpCAM as a better biomarker for diagnosing cHCC-ICC.

Whether tumor progression follows gradual or punctuated evolution is controversial (152). Guo et al. performed scDNA-seq and scRNA-seq on 28,975 cells from 14 patients with HCC, proposing a novel dual-phase copy number evolution (DPCNE) model (125). Accumulation of Copy number alterations (CNAs), elicited by genome instability, plays a pivotal role in hepatocarcinogenesis. They assessed CNA profiles of each cell, revealing that both punctuated copy number evolution (PCNE) and gradual copy number evolution (GCNE) coexisted in HCC. Subsequent mathematical analysis demonstrated that DPCNE model outperformed both GCNE model and PCNE model. Therefore, this research showed that punctuated evolution and gradual evolution are not mutually exclusive. Instead, both evolution patterns can coexist in the same tumor and may drive hepatocarcinogenesis at different stages. This discovery makes a crucial breakthrough in the oncogenesis of HCC, contributing to the clearer understanding of tumor progression.

### 3.4 Mechanisms of Metastasis in Liver Cancer

As an important biological behavior, metastasis is one of the hallmarks of cancer (154). CTCs are tumor cells that are shed from primary tumors and become metastatic deposits in the blood stream (155). The count and dynamic monitoring of CTCs can be used to evaluate the therapeutic effects of different agents and patient prognosis. However, further applications of CTCs in clinical diagnosis and treatment are limited by only counting CTCs and ignoring the genomic characteristics and heterogeneity of CTCs. Single-cell sequencing can be used to analyze the genome and transcriptome of CTCs, thus, clarifying the mechanisms of tumor metastasis and discovering new CTC biomarkers.

Substantial researches focus on the temporal heterogeneity in CTC phenotypes during anticancer treatments. However, the spatial heterogeneity of CTCs within anatomically distinct regions of the human circulatory system has been severely neglected. Sun et al. used scRNA-seq based on CTCs isolated from the peripheral veins and arteries, hepatic veins, infrahepatic inferior venae cavae, and portal veins of patients with HCC to demonstrate a profound spatial heterogeneity in cellular distribution and biological features which exists among CTCs (127). These CTCs were classified into three subsets (epithelial, intermediate, or mesenchymal) based on their EMT-related gene expression profile. CTCs of the epithelial subset mainly came from hepatic veins while the CTCs of the intermediate and mesenchymal subsets mostly came from peripheral veins, which suggested that mutations in blood CTCs were accumulated continually with the changes in the microenvironment, resulting in the heterogeneity of CTCs.

In another study of the same research team, scRNA-seq was applied to prove a previously unappreciated spatial heterogeneity and an immune-escape mechanism of CTCs based on 113 single CTCs from 4 different vascular sites (128). They examined the gene profiles of CTCs that specifically expressed in each vascular site, suggesting remarkable intravascular and intervascular heterogeneity in single CTCs from different vascular compartments. Notably, results of scRNA-seq demonstrated that CTCs developed a variety of immune-evasion strategies, including EMT, platelet-CTC aggregates, and the production of immunosuppressive chemokines. Among these methods, chemokine CCL5 was identified as the top differentially upregulated transcriptome related to immune evasion, indicating its significance in immune-escape mechanism of CTCs. Further analysis revealed that CCL5 promotes the metastatic potential of CTCs *via* recruiting Tregs during hematogenous transportation and Treg-derived TGF-β1 induced CCL5 production *via* p38-MAX signaling in turn. Their unique discovery of the CTC-CCL5-Tregs axis explains why CTCs can successfully escape from immune-mediated killing after leaving the protective immunosuppressive TME. Additionally, blocking CCL5 or CCR5 can be designed as promising anti-metastasis therapeutic strategies in HCC. Targeting CCL5 or CCR5 may provide an opportunity to eradicate CTCs within blood vessels, preventing their arrival at distant organs. This may effectively hinder the distant metastasis of HCC.

## 4 Discussion and Perspective

Intratumor heterogeneity which plays a key role in diagnosis, treatment response, disease progression, and survival outcomes, is a typical feature of liver cancers. Since the tumor tissue is a mixture of various cells, traditional analyses of tumor tissue usually show the average level of cell population, ignoring the intratumor heterogeneity. In recent years, single-cell sequencing has enabled analysis of the gene expression profiles at the single-cell level and provides novel insights for cancer precision medicine. High throughput sequencing of tumor cells, CSCs, single cells in TMEs, and CTCs can reveal the heterogeneity of liver cancer cells and their immune microenvironment, which allows more in-depth research on the occurrence, progression, and metastasis of liver cancers and provide more suitable biomarkers for precision medicine, including clinical diagnosis, individualized treatment, efficacy evaluation, and prognosis. The landscape of TMEs in liver cancers has been revealed by single-cell sequencing gradually, and this may contribute to the more precise immunotherapy of liver cancers.

Among all single-cell sequencing technologies, scRNA-seq is the most advanced and widely used in liver cancer studies due to relatively higher throughput and lower cost than the other single-cell sequencing methods. It has also been used in non-coding RNA studies. Through scRNA-seq of circular RNA, circASAP1 was identified as a significant regulator of HCC metastasis and serves as a prognostic predictor in patients with HCC (156). Furthermore, single-cell genomic and epigenomic sequencing technologies have also been gradually developed and scTrio-seq has already been applied in a heterogeneity study in HCC that reveals the mechanism by which the transcriptome, genome, and DNA methylome regulate each other (76). Of note, single-cell VCS has been used in HBV-associated HCC studies (123). In contrast, single-cell proteomics is rarely applied in liver cancer studies because of the extremely low copy number of individual proteins and the lack of amplification methods. Similarly, the use of single-cell histone modification sequencing and single-cell chromatin structure sequencing is not widespread.

Most of the above-mentioned studies capture single cells from less than 15 patients, perhaps due to the limitations of cost. However, more patients should be included in future studies to analyze the connection between the data from single-cell sequencing and patient outcomes. In addition to this, few researches are focused on multiple aspects of liver cancer to date. Therefore, studies that cover multiple distinct aspects might be more convincing. In the meantime, since a growing number of cells are used for single-cell sequencing, more powerful bioinformatics technologies are needed to identify subtle distinctions (in databases) that reflect cell states.

Our understanding of tumor biology has been significantly advanced, on account of the development and applications of single-cell technologies. Some fundamental questions in liver cancer can be explored by single-cell sequencing. First, intricate ecosystem poses significant challenges to precision medicine in liver cancer, in view of the highly heterogeneous cancer cell population both between tumors and within a tumor. Applications of these powerful single-cell technologies make it possible to search for the key to the creation of such a sophisticated ecosystem. Subsequently, therapeutic strategies designed to target the pivotal cells or factors in liver cancer may contribute to the individualized medicine. Second, targeted therapy and immunotherapy are transforming the treatment approach for liver cancer. Single-cell sequencing technologies have been used to detect the response of immune checkpoint therapy in cutaneous basal cell carcinoma and melanoma (91, 157). Recently, a multiomic analysis has been applied to reveal the impacts of intertumor heterogeneity on patient response to targeted therapy and immunotherapy in multifocal ICC (158). However, no similar study has been performed for patients with HCC to date. Such studies need to be applied in the field of HCC to help optimize patient selection for targeted therapy or immunotherapy, predict the prognosis, treatment response, and treatment resistance. Finally, analysis of CTCs holds great potential to be a noninvasive solution for clinical cancer management. A novel integrated system has been developed for the detection and downstream single-cell analysis of CTCs (159). Heterogeneity of CTCs can be unveiled by precise, highly automated single CTC enumeration and molecular characterization. Single-cell sequencing of CTCs will not only be useful for studying tumor evolution and dissemination but will also be a powerful tool to enable the personalized therapeutics of liver cancer.

## 5 Conclusion

In conclusion, single-cell sequencing is a rapidly developing technology that enables the analysis of genomic, transcriptomic, and epigenetic information at the single-cell level and provides novel insights for cancer precision medicine (6). However, several challenges need to be solved before single-cell sequencing can be widely used in clinics. For instance, the cost of single-cell sequencing is so expensive that the detection of a large number of cells is unaffordable for most patients. Consequently, only a few cells are used for single-cell sequencing at any given time. In addition, the data of single-cell sequencing is rarely associated with clinical outcomes. Understanding how heterogeneity, TMEs, and tumor evolution contribute to clinically relevant outcomes from the aspect of a single cell may be a promising potential application of this sequencing technology and can promote the development of precision medicine. Moreover, the detection of CTCs depends on the analysis of only a small number of surface markers, which makes the isolation of CTCs difficult. In fact, the only device that FDA approves to detect CTCs is based on the expression of EpCAM (160). Nevertheless, this CTC assay is unable to isolate CTCs from de-differentiated tumors and carcinomas undergoing loss of this epithelial phenotype during the process of invasion and metastasis (161). As a result, numerous teams of researchers are developing many different methods for CTC detection (161, 162). These technologies that have the capability to efficiently isolate and evaluate CTCs in a sensitive manner would broaden the potential applications of non-invasive liquid biopsies of patients with PLCs and single-cell sequencing of CTCs.

Inspiringly, the solutions of reducing costs, shortening sequencing time, enhancing the sensitivity and specificity of sequencing, improving the coverage and accuracy of amplification, and finding more suitable single cell markers are forthcoming due to the continuous optimization of amplification methods and the rapid development of bioinformatics. Therefore, it is undoubtedly reasonable to foresee that single-cell sequencing will be applied in numerous novel fields of liver cancer research in the near future.

## Author Contributions

BT performed the literature research, and drafted the manuscript. QL revised the manuscript and gave the final approval for the article to be published. All authors read and approved the final manuscript.

## Funding

This work was supported by the National Natural Science Foundation of China (grant NO.82073214), Outstanding disciplines leaders of Shanghai Municipal Commission of Health and Family Planning (NO. 2018BR39) and Bethune Charitable Foundation.

## Conflict of Interest

The authors declare that the research was conducted in the absence of any commercial or financial relationships that could be construed as a potential conflict of interest.

## Publisher’s Note

All claims expressed in this article are solely those of the authors and do not necessarily represent those of their affiliated organizations, or those of the publisher, the editors and the reviewers. Any product that may be evaluated in this article, or claim that may be made by its manufacturer, is not guaranteed or endorsed by the publisher.

## Abbreviations

PLC, Primary liver cancer; TME, Tumor microenvironment; HCC, Hepatocellular carcinoma; ICC, Intrahepatic cholangiocarcinoma; CSCs, Cancer stem cells; CTCs, Circulating tumor cells; LCM, Laser capture microdissection; FACS, Fluorescence-activated cell sorting; MACS, Magnetic-activated cell sorting; EpCAM, Epithelial cell adhesion molecule; FDA, Food and Drug Administration; WGA, Whole genome amplification; PCR, Polymerase chain reaction; CNVs, Copy-number variations; DOP-PCR, Degenerate oligonucleotide-primed polymerase chain reaction; MDA, Multiple displacement amplification; MALBAC, Multiple annealing and looping-based amplification cycles; STRT-seq, Single-cell tagged reverse transcription sequencing; Smart-seq, Switching mechanism at 5’ end of the RNA transcript sequencing; CEL-seq, Cell expression by linear amplification and sequencing; RRBS, Reduced representation bisulfite sequencing; WGBS, Whole genome bisulfite sequencing; CGI-seq, CpG island methylation sequencing; ATAC-seq, Assay for transposase accessible chromatin sequencing; Trio-seq, Triple omics sequencing; RNA-seq, RNA sequencing; DPT cells, Double-positive T cells; TCR-seq, T-cell receptor sequencing; DNA-seq, DNA sequencing; WGS, whole-genome sequencing;

cHCC-ICC, Combined hepatocellular and intrahepatic cholangiocarcinoma; VCS, Virome capture sequencing; DEGs, Differentially expressed genes; CTSE, Cathepsin E; EMT, Epithelial-mesenchymal transition; SPINK1, Serine protease inhibitor Kazal-type 1; Tregs, Regulatory T cells; VEGF, Vascular endothelial growth factor;

CAFs, Cancer-associated fibroblasts; TAMs, Tumor-associated macrophages;

TECs, Tumor-associated endothelial cells; vCAFs, Vascular cancer-associated fibroblasts; HBV, Hepatitis B virus; PVTT, Portal vein tumor thrombus; DPCNE, Dual-phase copy number evolution; CNAs, Copy number alterations; GCNE, Gradual copy number evolution; PCNE, Punctuated copy number evolution.

## Glossary

**Table:**

PLC	Primary liver cancer
TME	Tumor microenvironment
HCC	Hepatocellular carcinoma
ICC	Intrahepatic cholangiocarcinoma
CSCs	Cancer stem cells
CTCs	Circulating tumor cells
LCM	Laser capture microdissection
FACS	Fluorescence-activated cell sorting
MACS	Magnetic-activated cell sorting
EpCAM	Epithelial cell adhesion molecule
FDA	Food and Drug Administration
WGA	Whole genome amplification
PCR	Polymerase chain reaction
CNVs	Copy-number variations
DOP-PCR	Degenerate oligonucleotide-primed polymerase chain reaction
MDA	Multiple displacement amplification
MALBAC	Multiple annealing and looping-based amplification cycles
STRT-seq	Single-cell tagged reverse transcription sequencing
Smart-seq	Switching mechanism at 5’ end of the RNA transcript sequencing
CEL-seq	Cell expression by linear amplification and sequencing
RRBS	Reduced representation bisulfite sequencing
WGBS	Whole genome bisulfite sequencing
CGI-seq	CpG island methylation sequencing
ATAC-seq	Assay for transposase accessible chromatin sequencing
Trio-seq	Triple omics sequencing
RNA-seq	RNA sequencing
DPT cells	Double-positive T cells
TCR-seq	T-cell receptor sequencing
DNA-seq	DNA sequencing
WGS	whole-genome sequencing
